# Paraplegia after Gastrectomy in a Patient with Cervical Disc Herniation: A Case Report and Review of Literature

**DOI:** 10.1155/2014/718690

**Published:** 2014-03-18

**Authors:** Qingfu Zhang, Wei Jiang, Quanhong Zhou, Guangyan Wang, Linlin Zhao

**Affiliations:** Department of Anesthesiology, Shanghai Jiao Tong University Affiliated Shanghai Sixth People's Hospital, 600 Yishan Road, Shanghai 200233, China

## Abstract

Paraplegia is a rare postoperative complication. We present a case of acute paraplegia after elective gastrectomy surgery because of cervical disc herniation. The 73-year-old man has the medical history of cervical spondylitis with only symptom of temporary pain in neck and shoulder. Although the patient's neck was cautiously preserved by using the Discopo, an acute paraplegia emerged at about 10 hours after the operation. Severe compression of the spinal cord by herniation of the C4-C5 cervical disc was diagnosed and emergency surgical decompression was performed immediately. Unfortunately the patient showed limited improvement in neurologic deficits even after 11 months.

## 1. Introduction

Paraplegia is a rare postoperative complication, and the pathology is various. We present a case of acute paraplegia after elective gastrectomy surgery because of cervical disc herniation. The IRB of Shanghai Sixth People's Hospital reviewed the case report and gave permission for us to publish the report.

## 2. Case Description

A 73-year-old man with peptic ulcer and bleeding was checked into the Department of Gastroenterology due to brown vomit and drain black stool once. The patient has a past medical history of duodenal ulcer for 18 years and complained from abdominal discomfort for 4 days. He received medical treatment with omepazole for 10 days and then was referred to the Department of General Surgery for selective gastrectomy. He denied any other medical history or other medication during preoperative visit by anesthetist.

General anesthesia was induced by intravenous administration of 15 *μ*g/kg fentanyl, 2 mg/kg propofol, and 0.1 mg/kg rocuronium. As the patient had loosened teeth, Discopo was taken for orotracheal intubation. During the whole process, the patient's neck was placed in a neutral position. The patient was mechanically ventilated with the settings of FiO_2_ 1.0, tidal volume 8 mL/kg, respiratory rate 10/min, and inspiration/expiration 1/2 and one minimum alveolar concentration of sevoflurane was administered during the surgery. In the meantime, propofol (2 mg/kg/h) and fentanyl (3 *μ*g/kg/h) were also infused.

**Figure 1:**
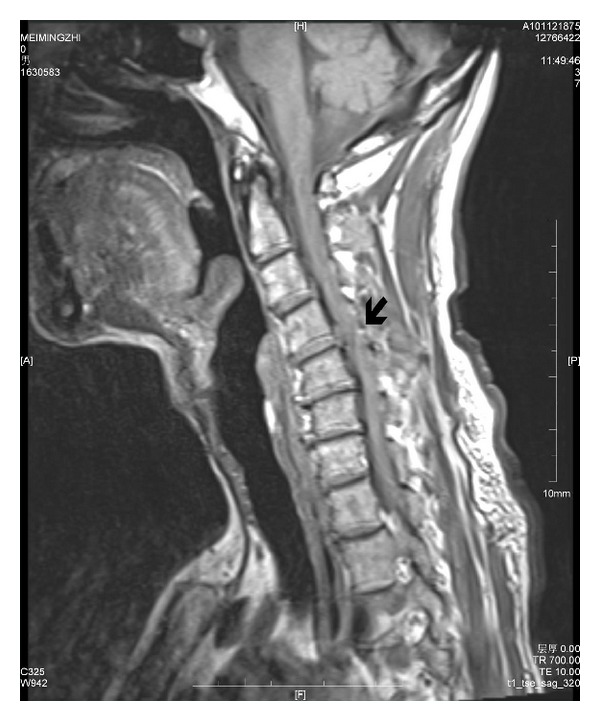
T2-weighted MRI demonstrates disc protrusion at C3-C4, C4-C5, and C5-C6 and corresponding spinal canal stenosis (preoperative MRI). Arrow shows the lesion at the C4-5 level before the operation.

**Figure 2:**
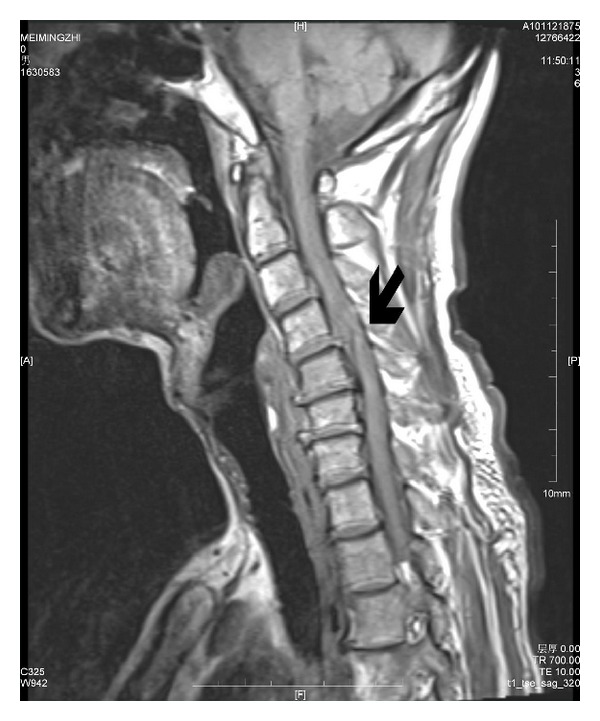
T2-weighted MRI demonstrates disc protrusion at C3-C4, C4-C5, and C5-C6, with spinal cord degeneration at 20 hours after gastrectomy. Arrow shows the lesion at the C4-5 level after the operation.

Subtotal gastrectomy was performed, and gastrointestinal tract was reconstructed with the method of Billroth II. The operation, which lasted about 2 hours, was uneventful with a total blood loss of 250 mL. There was no hemodynamic instability during surgery. The patient was sent to the postoperative care unit (PACU) and extubated 30 minutes later. The recovery process was smooth, and the patient was transferred to surgery intensive care unit (SICU). Ten hours after the arrival at SICU, the patient was found to be flaccid in both his legs. Neurological examinations revealed complete paralysis of the bilateral lower extremities, bilateral weakness of upper extremities, and absence of deep and superficial sensation below T4 level. CT scan was negative for intracranial lesions. Further, when asked about previous medical history, he said he had cervical spondylosis before, with the only symptom of intermittent pain in neck and shoulder. A preoperative MRI showed a protruded intervertebral disc between C3 and C4, C4 and C5, C5 and C6, and corresponding spinal canal stenosis (Figure 1). Repeated MRI of the neck at 20 hours after gastrectomy demonstrated a posterior disc herniation at C4-5, and spinal canal stenosis from C3 to C6, with spinal cord degeneration (Figure 2). After consultation with neurosurgeons, an emergency anterior approach to the C3–C5 vertebral disectomy was performed immediately, followed by C4 vertebral resection and interbody fusion with iliac crest bone graft. After the surgery, the patient's upper extremities improved, however, lower extremities remained paralyzed without significant improvement at 11 months of follow up.

## 3. Discussion 

Nontraumatic paraplegia caused by cervical disc herniation is rare. Since it was first reported in 1973 [1], more cases have been described in detail [2–12], especially in the last decade (Table 1). We present postoperative paraplegia due to acute compression of spinal cord secondary to the protrusion of cervical disc.

Nontraumatic paraplegia is an emergent condition. It is difficult to make an accurate diagnosis if there was no obvious injury. Paraplegia caused by cervical disc herniation after general anesthesia was even more rare. The possible etiology of cervical herniation during operation was not clear. Various etiologies should be kept in mind, such as intracranial lesions, spinal infarction, disorders of muscle, or neuromuscular junctions [9]. For this case, CT scan of the brain was negative. The patient denied any disorders of muscle or neuromuscular junctions.

Among our review of the literature, there are 5 cases that had apparent cause for the onset of paraplegia. Two developed following bending forward to do something [9, 12], 1 following being fixed to the headrest of the MRI instrument [10], 1 following rolling in bed on the left side [4], and 1 is in association with labor [7]. These events may have led to the posterior movement of the disk, which in turn caused herniation, increasing the compression on the dural sac and leading to severe mechanical compression and ischemia of the spinal cord [10]. It is likely that extension of the cervical spine loosened the tension of the posterior longitudinal ligament and caused posterior listhesis of the vertebra.

Excessive neck extension in intubation and changing the position of neck during general anesthesia and loss of muscle support may aggravate spinal cord injury [5]. Coexisting cervical spine disorders, such as spondylosis, bulging disc, and spinal canal stenosis, are not uncommon in the elderly patients, which raises the possibility of spinal cord injury during general anesthesia. In our case, the medical history of cervical spondylosis and previous MRI test led us to suspect a lesion within the cervical cord.

The incidence rate in Asians is higher than others. This could be explained by the narrower spinal canal anatomy among Asians [9, 13, 14]. In the present case, the patient had spinal canal stenosis which made him more vulnerable to compressive disturbances [15, 16]. To make things worse, the patient did not mention his previous cervical spondylosis history. His previous MRI was overlooked by all medical staff before the gastrectomy. Although careful history taking was performed, details not shared with medical staff might cause serious consequences as in this case.

There are many techniques of intubation commonly used for patients with cervical spondylosis such as awake endotracheal intubation, flexible fiberoptic bronchoscope, and Discopo [17]. Unfortunately, complications still happened. Deem et al. [2] reported quadriplegia after thoracolumbar surgery in a patient with severe cervical spondylosis, despite the fact that awake oral tracheal intubation under direct visual laryngoscopy was performed without difficulty. Hwang et al. [8] described quadriparesis after CABG in a patient with a history of cervical spondylosis although all precautions to prevent hyperextension of the neck during intubation and patient positioning were considered. In the present case, although Discopo was used to minimize the movement of the neck and to keep the head in neutral position, for endotracheal intubation, the cervical herniation still happened. The lesson from the case is to keep the neck support for cervical spondylosis during noncervical surgery. We also suggest that, in patients with significant cervical diseases undergoing elective noncervical spine surgery, cervical decompression should be considered as the initial treatment.

In conclusion, paraplegia after noncervical spine surgery under general anesthesia is a devastating complication, which often results in permanent disability or neurological deficit in patients with preexisting cervical spine diseases. Anesthesiologists and surgeons should pay much attention to this complication and share details of history with each other in order to exclude the coexisting cervical spine disorders. Excessive neck movement is believed to be trigging factor and skillful intubation and neck supporting are recommended to reduce spinal cord injury.

## Figures and Tables

**Table 1:** Reported cases of nontraumatic acute myelopathy due to cervical disc herniation.

Authors	Year	Country	Age/sex	After general anesthesia	level	Spinal stenosis	Operation	Recovery of motor function
Lourie et al. [1]	1973	USA	37/M	−	C6-C7	−	ASF	+
Kawaguchi et al. [18]	1991	USA	61/M	+	C6-C7	−	ASD	+
Ueyama et al. [3]	1999	Japan	61/F	−	C6-C7	+	ASF	+
Suzuki et al. [4]	2003	Japan	29/M	−	C6-C7	+	ASF	−
Chen et al. [5]	2005	Taiwan	54/M	+	C6-C7	−	ASF	−
Hirose and Akhrass [6]	2005	USA	65/M	+	C7-T1	−	ASF	−
Tsai et al. [7]	2006	Taiwan	32/F	−	C3-c4	−	ASF	+
Hwang et al. [8]	2008	Singapore	63/M	+	C5-C6, C6-C7	+	No operation	+
Liu et al. [9]	2010	China	75/M	−	C4-C5	+	ASF	−
Gorur et al. [19]	2010	Turkey	62/M	+	C5-C6	−	ASF	+
Kato et al. [10]	2010	Japan	48/M	−	C6-C7	−	ASF	+
Ikeda et al. [11]	2012	Japan	21/F	−	C3-C4	+	ASF	+
Ahmed et al. [12]	2013	Egypt	48/F	−	C5-C6	+	ASF	+
Present case	2013	China	73/M	+	C4-C5	+	ASF	−

M: male; F: female; ASF: anterior spine fusion; ASD: anterior spine decompression.
